# Supplementary material for: Nec‐1 alleviates cognitive impairment with reduction of Aβ and tau abnormalities in APP/PS1 mice
Source: EMBO Mol Med. 2016 Nov 17;9(1):61–77. doi: 10.15252/emmm.201606566 (PMC5210088; doi:10.15252/emmm.201606566)
Supplement: Supplementary file 1 — Table EV1 [file EMMM-9-61-s001.docx]

**Table EV1.** Statistical analyses of MTT cytotoxicity assay and Live/Dead Viability/Cytotoxicity assay for Fig 1B, C and E. All data presented in this article are representative results of at least three independent experiments.

**A. Statistical analyses of MTT cytotoxicity assay in Fig 1B.**

| NT vs. Aβ(1-42) 10 μM, *p* = 0.0018  Nec-1 10 μM vs. Aβ(1-42) 10 μM, *p* = 0.0001  Nec-1 25 μM vs. Aβ(1-42) 10 μM, *p* = 0.0001  Nec-1 50 μM vs. Aβ(1-42) 10 μM, *p* = 0.0151  Nec-1 100 μM vs. Aβ(1-42) 10 μM, *p* = 0.0006  Nec-1 200 μM vs. Aβ(1-42) 10 μM, *p* = 0.0018 |
| --- |

**B. Statistical analyses of MTT cytotoxicity assay in Fig 1C.**

| **HT22**  **12h** Aβ(1-42) vs. Aβ(1-42) + Nec-1, *p* = 0.002  **24h** Aβ(1-42) vs. Aβ(1-42) + Nec-1, *p* = 0.002  **BV2**  **12h** Aβ(1-42) vs. Aβ(1-42) + Nec-1, *p* = 0.0004  **24h** Aβ(1-42) vs. Aβ(1-42) + Nec-1, *p* = 0.0109  **Primary Astrocytes**  **12h** Aβ(1-42) vs. Aβ(1-42) + Nec-1, *p* = 0.0002  **24h** Aβ(1-42) vs. Aβ(1-42) + Nec-1, *p* = 0.0003 |
| --- |

**C. Statistical analyses of Live/Dead Viability/Cytotoxicity assay in Fig 1E.**

| NT vs. Aβ(1-42), *p* = 0.0024  Aβ(1-42) vs. Aβ(1-42) + Nec-1, *p* = 0.0014 |
| --- |
