# Supplementary material for: Nec‐1 alleviates cognitive impairment with reduction of Aβ and tau abnormalities in APP/PS1 mice
Source: EMBO Mol Med. 2016 Nov 17;9(1):61–77. doi: 10.15252/emmm.201606566 (PMC5210088; doi:10.15252/emmm.201606566)
Supplement: Supplementary file 2 — Table EV2 [file EMMM-9-61-s002.docx]

**Table EV2.** Statistical analyses of Y-maze and Passive avoidance test after Nec-1 administration for Fig 2B-D and 2H-I.

**A. Statistical analyses of Y-maze test in Fig 2B.**

| Wt (Veh) vs. Wt (Nec-1), *p* = 0.9780  APP/PS1 (Veh) vs. APP/PS1 (Nec-1), *p* = 0.0146  Wt (Veh) vs. APP/PS1 (Veh), *p* = 0.0014  Wt (Nec-1) vs. APP/PS1 (Nec-1), *p* = 0.4324 |
| --- |

**B. Statistical analyses of Y-maze test in Fig 2C.**

| Wt (Veh) vs. Wt (Nec-1), *p* = 0.7053  APP/PS1 (Veh) vs. APP/PS1 (Nec-1), *p* = 0.6122  Wt (Veh) vs. APP/PS1 (Veh), *p* = 0.1074  Wt (Nec-1) vs. APP/PS1 (Nec-1), *p* = 0.1790 |
| --- |

**C. Statistical analyses of Passive avoidance test in Fig 2D.**

| **Acquisition**  Wt (Veh) vs. Wt (Nec-1), *p* = 0.8325  APP/PS1 (Veh) vs. APP/PS1 (Nec-1), *p* = 0.9132  Wt (Veh) vs. APP/PS1 (Veh), *p* = 0.7354  Wt (Nec-1) vs. APP/PS1 (Nec-1), *p* = 0.3202  **Retention**  Wt (Veh) vs. Wt (Nec-1), *p* = 0.1469  APP/PS1 (Veh) vs. APP/PS1 (Nec-1), *p* = 0.0429  Wt (Veh) vs. APP/PS1 (Veh), *p* = 0.0036  Wt (Nec-1) vs. APP/PS1 (Nec-1), *p* = 0.3432 |
| --- |

**D. Statistical analyses of Y-maze test in Fig 2H.**

| Veh vs. Nec-1, *p* = 0.5284  Aβ(1-42) vs. Aβ(1-42) + Nec-1, *p* = 0.0002  Veh vs. Aβ(1-42), *p* = 0.0004  Nec-1 vs. Aβ(1-42) + Nec-1, *p* = 0.1807 |
| --- |

**E. Statistical analyses of Y-maze test in Fig 2I.**

| Veh vs. Nec-1, *p* = 0.7021  Aβ(1-42) vs. Aβ(1-42) + Nec-1, *p* = 0.9547  Veh vs. Aβ(1-42), *p* = 0.1561  Nec-1 vs. Aβ(1-42) + Nec-1, *p* = 0.1407 |
| --- |
