# Supplementary material for: Nec‐1 alleviates cognitive impairment with reduction of Aβ and tau abnormalities in APP/PS1 mice
Source: EMBO Mol Med. 2016 Nov 17;9(1):61–77. doi: 10.15252/emmm.201606566 (PMC5210088; doi:10.15252/emmm.201606566)
Supplement: Supplementary file 3 — Table EV3 [file EMMM-9-61-s003.docx]

**Table EV3.** Statistical analyses of total numbers and areas of plaques in the brains of APP/PS1 mice after vehicle or Nec-1 administration for Fig 4B-G.

**A. Statistical analyses of total plaque numbers in Fig 4B.**

| APP/PS1 (Veh) vs. APP/PS1 (Nec-1), *p* = 0.000008 (*p* < 0.0001) |
| --- |

**B. Statistical analyses of total plaque areas in Fig 4C.**

| APP/PS1 (Veh) vs. APP/PS1 (Nec-1), *p* = 0.0012 |
| --- |

**C. Statistical analyses of total plaque numbers in Fig 4D.**

| APP/PS1 (Veh) vs. APP/PS1 (Nec-1), *p* = 0.000012 (*p* < 0.0001) |
| --- |

**D. Statistical analyses of total plaque areas in Fig 4E.**

| APP/PS1 (Veh) vs. APP/PS1 (Nec-1), *p* = 0.0038 |
| --- |

**E. Statistical analyses of total plaque numbers in Fig 4F.**

| APP/PS1 (Veh) vs. APP/PS1 (Nec-1), *p* = 0.00003 (*p* < 0.0001) |
| --- |

**F. Statistical analyses of total plaque areas in Fig 4G.**

| APP/PS1 (Veh) vs. APP/PS1 (Nec-1), *p* = 0.0016 |
| --- |
