# Supplementary material for: Nec‐1 alleviates cognitive impairment with reduction of Aβ and tau abnormalities in APP/PS1 mice
Source: EMBO Mol Med. 2016 Nov 17;9(1):61–77. doi: 10.15252/emmm.201606566 (PMC5210088; doi:10.15252/emmm.201606566)
Supplement: Supplementary file 4 — Table EV4 [file EMMM-9-61-s004.docx]

**Table EV4.** Statistical analyses of dot blot analyses, Aβ-soluble and -insoluble levels in the brains of APP/PS1 mice after vehicle or Nec-1 administration, and ThT assay for Fig 5D, E and G, respectively. All data presented in this article are representative results of at least two independent experiments.

**A. Statistical analyses of total Aβ and protein oligomer in Fig 5D.**

| **Cortex**  **Aβ (6E10)** Wt (Veh) vs. APP/PS1 (Veh), *p* = 0.0500  APP/PS1 (Veh) vs. APP/PS1 (Nec-1), *p* = 0.3315  Wt (Veh) vs. APP/PS1 (Nec-1), *p* = 0.0237  **Oligomer (A11)** Wt (Veh) vs. APP/PS1 (Veh), *p* = 0.0018  APP/PS1 (Veh) vs. APP/PS1 (Nec-1), *p* = 0.0089  Wt (Veh) vs. APP/PS1 (Nec-1), *p* = 0.3842  **Hippocampus**  **Aβ (6E10)**  Wt (Veh) vs. APP/PS1 (Veh), *p* = 0.0461  APP/PS1 (Veh) vs. APP/PS1 (Nec-1), *p* = 0.3866  Wt (Veh) vs. APP/PS1 (Nec-1), *p* = 0.0500 |
| --- |

**B. Statistical analyses of Aβ-soluble and –insoluble fractions in Fig 5E.**

| **Cortex**  **Soluble** Wt (Veh) vs. APP/PS1 (Veh), *p* = 0.0017  APP/PS1 (Veh) vs. APP/PS1 (Nec-1), *p* = 0.0014  Wt (Veh) vs. APP/PS1 (Nec-1), *p* = 0.9613  **Insoluble** Wt (Veh) vs. APP/PS1 (Veh), *p* = 0.0001  APP/PS1 (Veh) vs. APP/PS1 (Nec-1), *p* = 0.0001  Wt (Veh) vs. APP/PS1 (Nec-1), *p* = 0.0100  **Hippocampus**  **Soluble** Wt (Veh) vs. APP/PS1 (Veh), *p* = 0.0053  APP/PS1 (Veh) vs. APP/PS1 (Nec-1), *p* = 0.0053  Wt (Veh) vs. APP/PS1 (Nec-1), *p* = 0.9734  **Insoluble** Wt (Veh) vs. APP/PS1 (Veh), *p* = 0.0002  APP/PS1 (Veh) vs. APP/PS1 (Nec-1), *p* = 0.0007  Wt (Veh) vs. APP/PS1 (Nec-1), *p* = 0.0092 |
| --- |

**C. Statistical analyses of ThT assay in Fig 5G.**

| **Day5**  Nec-1(-) vs. Nec-1(+), *p* = 0.0007 |
| --- |
