# Supplementary material for: Nec‐1 alleviates cognitive impairment with reduction of Aβ and tau abnormalities in APP/PS1 mice
Source: EMBO Mol Med. 2016 Nov 17;9(1):61–77. doi: 10.15252/emmm.201606566 (PMC5210088; doi:10.15252/emmm.201606566)
Supplement: Supplementary file 5 — Table EV5 [file EMMM-9-61-s005.docx]

**Table EV5.** Statistical analyses of ThT assay for Fig 6D and E. All data presented in this article are representative results of at least three independent experiments.

**A. Statistical analyses of ThT assay in Fig 6D.**

| **Day5**  Nec-1(-) vs. Nec-1(+), *p* = 0.0007 |
| --- |

**B. Statistical analyses of ThT assay in Fig 6E.**

| **Aggregates**  Nec-1(-) vs. Nec-1(+), *p* = 0.0002 |
| --- |
