# Supplementary figures and images for: Nec‐1 alleviates cognitive impairment with reduction of Aβ and tau abnormalities in APP/PS1 mice
Source: EMBO Mol Med. 2016 Nov 17;9(1):61–77. doi: 10.15252/emmm.201606566 (PMC5210088; doi:10.15252/emmm.201606566)

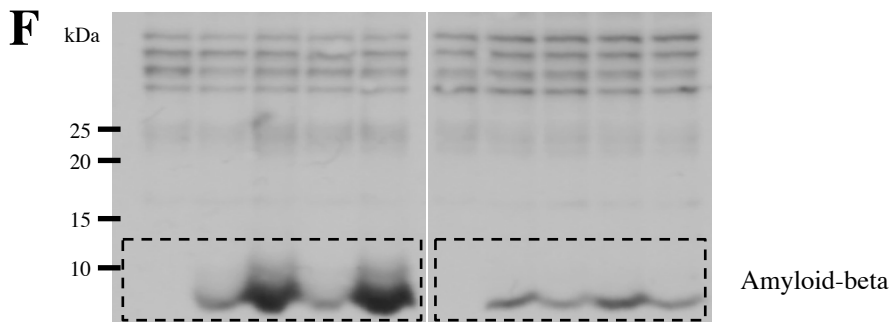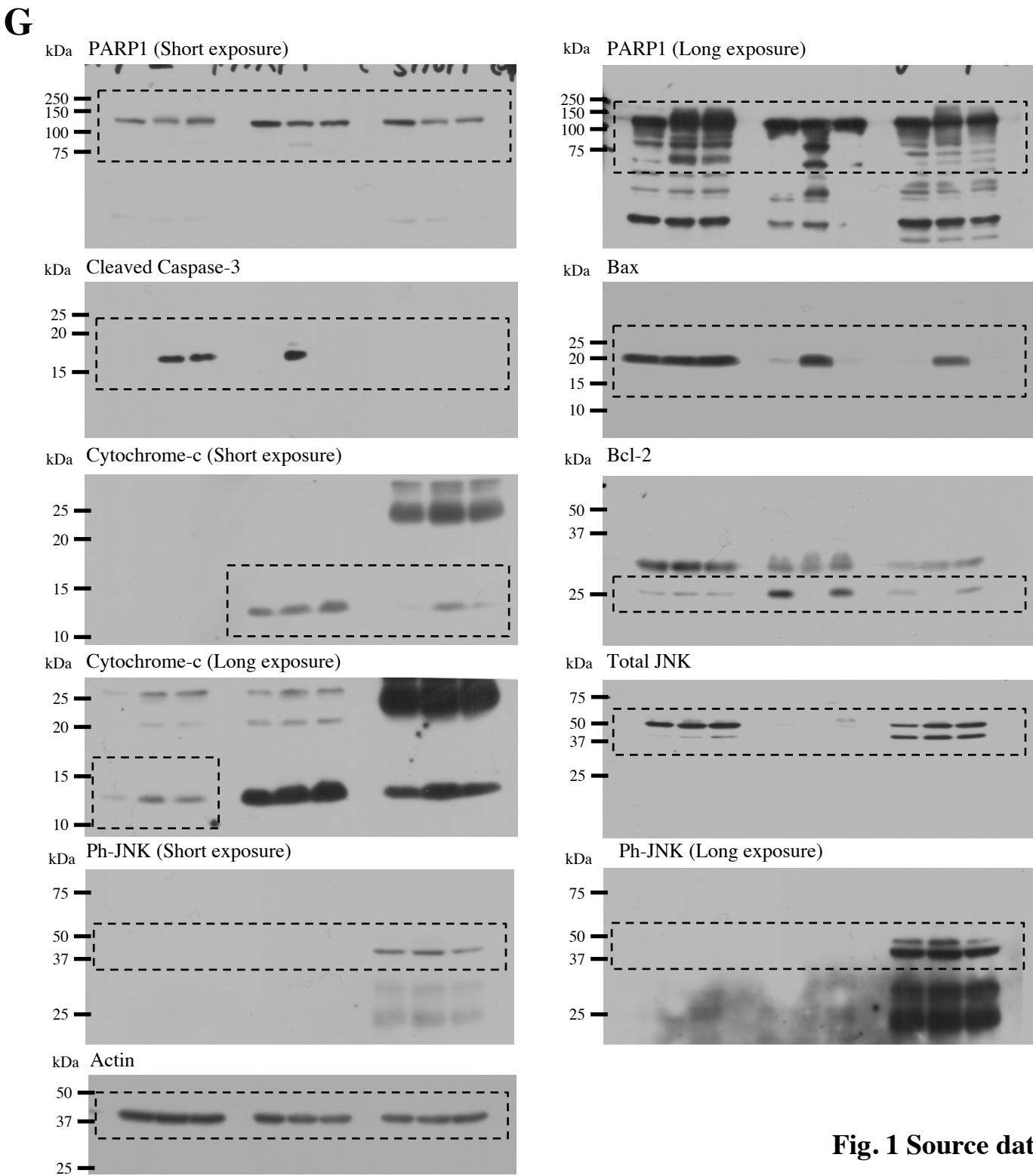

**Fig. 1 Source data**

Supplement: Supplementary file 7 — Source Data for Figure 1 [file EMMM-9-61-s006.pdf]

**A**

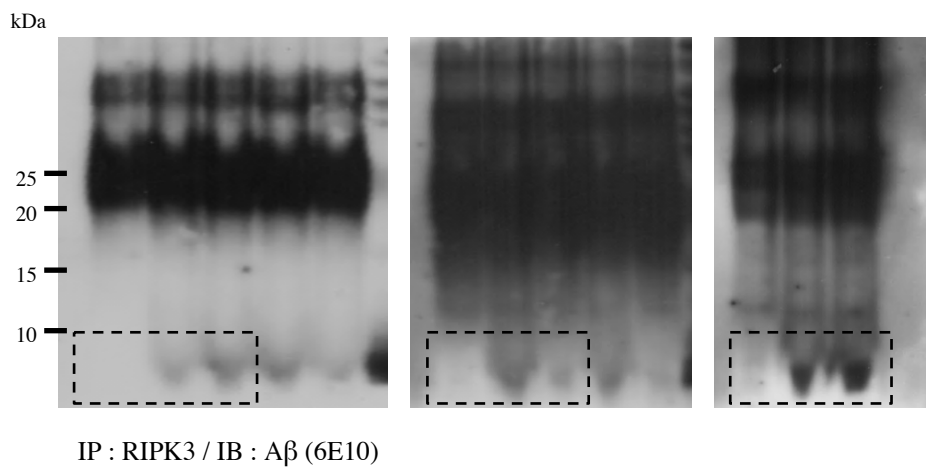

**Fig. 3 Source data**

Supplement: Supplementary file 8 — Source Data for Figure 3 [file EMMM-9-61-s007.pdf]

**F**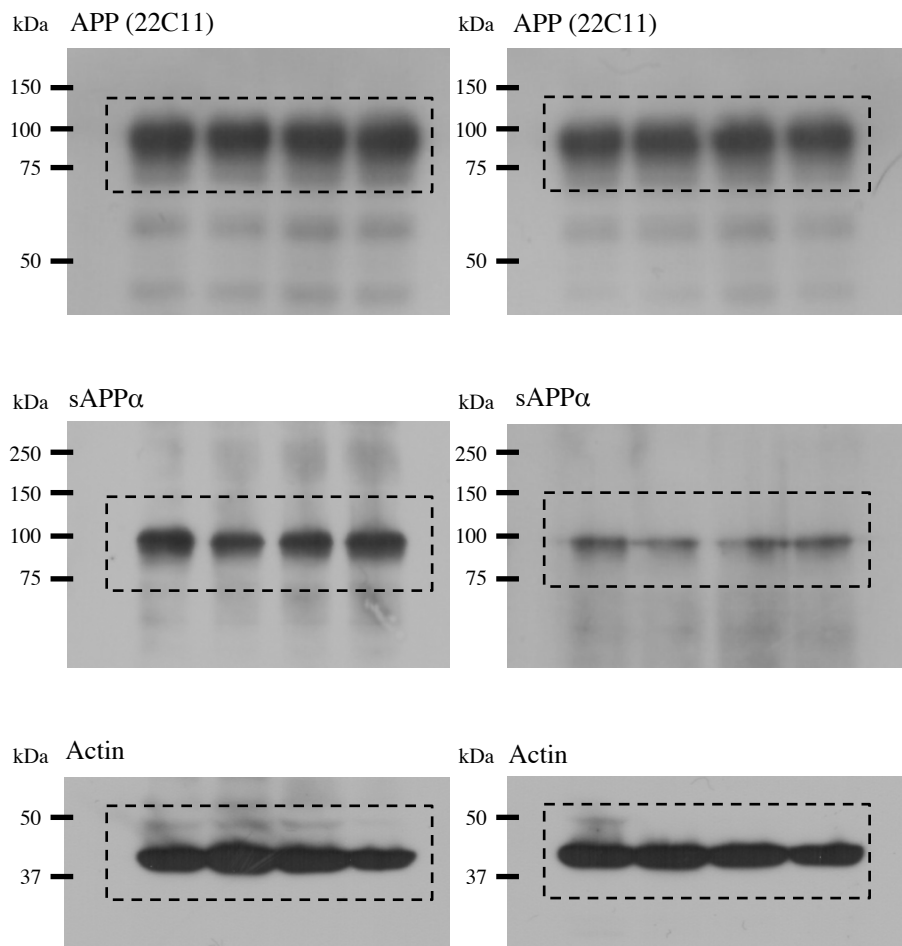**Fig. 5 Source data**

Supplement: Supplementary file 9 — Source Data for Figure 5 [file EMMM-9-61-s008.pdf]

**A**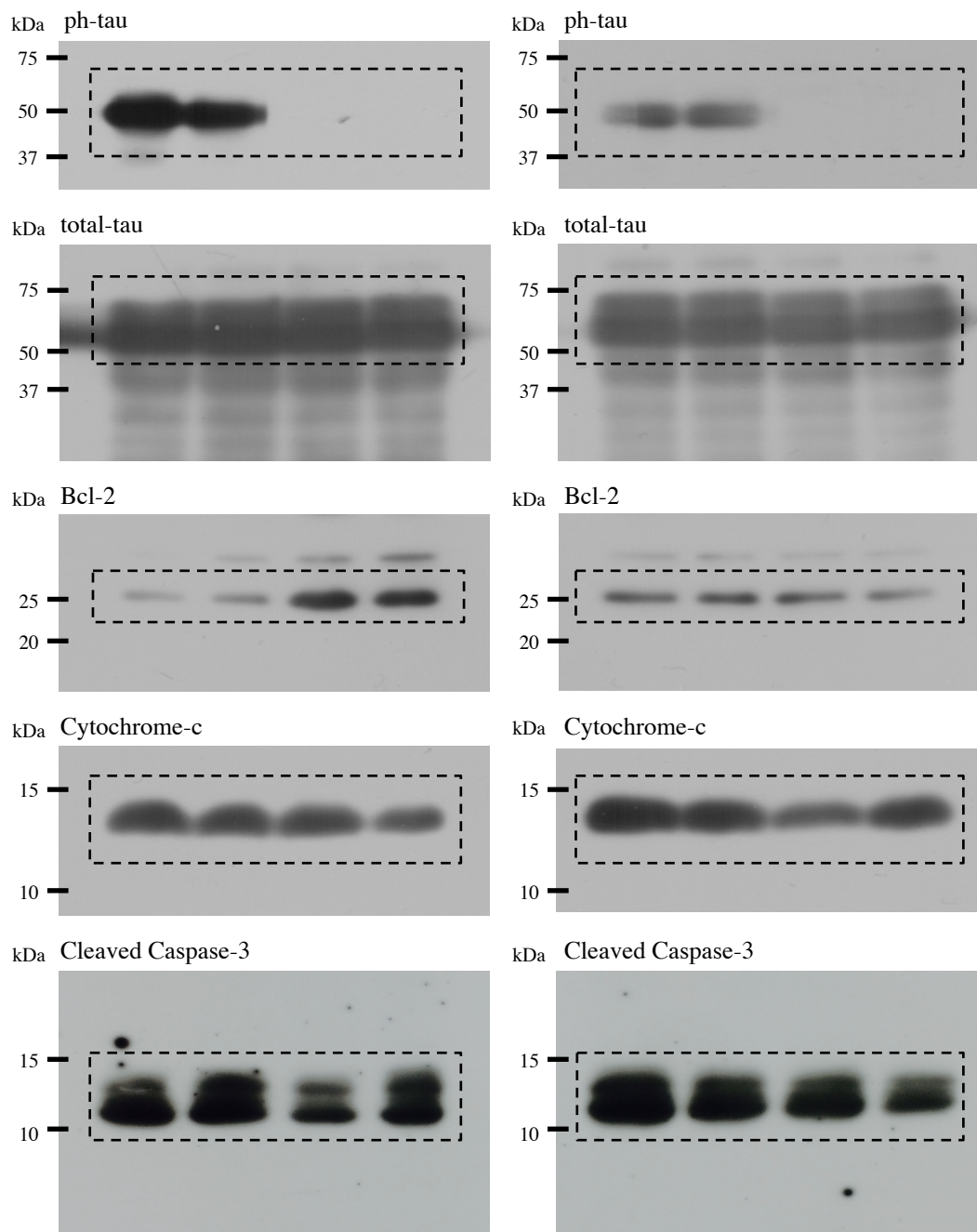**C**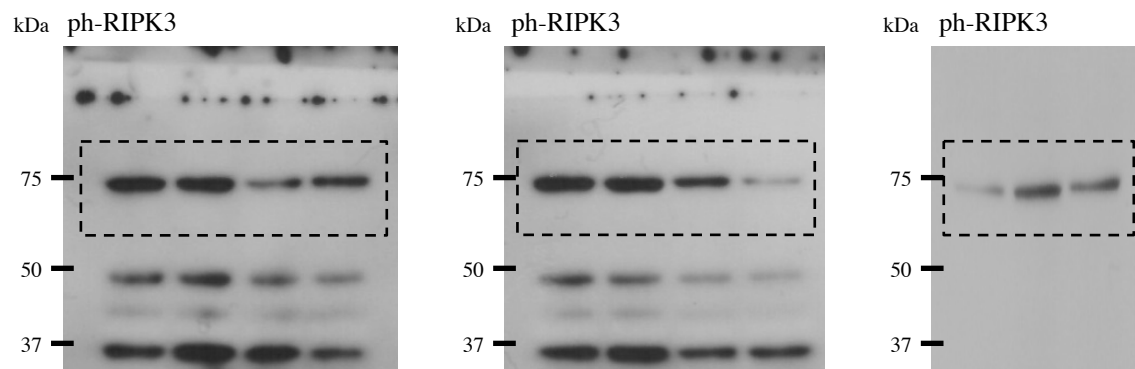**Fig. 6 Source data**

Supplement: Supplementary file 10 — Source Data for Figure 6 [file EMMM-9-61-s009.pdf]
